# Charge Transfer Complexes of 1,3,6-Trinitro-9,10-phenanthrenequinone with Polycyclic Aromatic Compounds

**DOI:** 10.3390/molecules26216391

**Published:** 2021-10-22

**Authors:** Roman Linko, Michael Ryabov, Pavel Strashnov, Pavel Dorovatovskii, Victor Khrustalev, Victor Davydov

**Affiliations:** 1 Faculty of Science, Peoples’ Friendship University of Russia (RUDN University), 6 Miklukho-Maklaya Street, 117198 Moscow, Russia; ryabov_ma@pfur.ru (M.R.); strashnov_pv@pfur.ru (P.S.); khrustalev_vn@pfur.ru (V.K.); davydov_vv@pfur.ru (V.D.); 2 Kurchatov Complex for Synchrotron and Neutron Investigations, National Research Center “Kurchatov Institute”, 123182 Moscow, Russia; paulgemini@mail.ru; 3 N.D. Zelinsky Institute of Organic Chemistry, Russian Academy of Sciences, 119991 Moscow, Russia

**Keywords:** charge transfer complex, DFT, X-ray diffraction, 1,3,6-trinitro-9,10-phenanthrenequinone, anthracene

## Abstract

Understanding the interactions of organic donor and acceptor molecules in binary associates is crucial for design and control of their functions. Herein, we carried out a theoretical study on the properties of charge transfer complexes of 1,3,6-trinitro-9,10-phenanthrenequinone (PQ) with 23 aromatic π-electron donors. Density functional theory (DFT) was employed to obtain geometries, frontier orbital energy levels and amounts of charge transfer in the ground and first excited states. For the most effective donors, namely, dibenzotetrathiafulvalene, pentacene, tetrathiafulvalene, 5,10-dimethylphenazine, and tetramethyl-p-phenylenediamine, the amount of charge transfer in the ground state was shown to be 0.134−0.240 e^−^. Further, a novel charge transfer complex of PQ with anthracene was isolated in crystalline form and its molecular and crystal structure elucidated by single-crystal synchrotron X-ray diffraction.

## 1. Introduction

Organic π-π charge transfer complexes (CTCs) form a special class of binary compounds stabilized by partial electron transfer between noncovalently interacting donor (D) and acceptor (A) molecules. The degree of electron transfer in CTCs is governed by the difference between the donor ionization potential and the acceptor electron affinity which can be approximated as the difference between the donor highest occupied molecular orbital (HOMO) and the acceptor lowest unoccupied molecular orbital (LUMO) [1]. The HOMO-LUMO energy gap can be obtained from DFT calculations.

Individual CTCs may undergo self-assembly and form crystalline or supramolecular structures [2]. The properties of such assemblies depend on the stoichiometric composition of the complexes [3,4] and their polymorphism [5,6]. CTCs in crystals tend to form one of two types of molecular stacks: (a) mixed-type stacks with alternating donor and acceptor molecules {-D-A-D-A}_∞_ or {-D-A-D-D-A-D}_∞_ and (b) segregated stacks of donor and acceptor molecules {-D-D-D-}_∞_ ǁ {-A-A-A-}_∞_ [3,7].

CTCs exhibit a wide range of physical properties therefore the search for new effective electron donors, acceptors, and synthesis of new CTCs on this basis is of high relevance [8]. At the same time, quantum-chemical modeling is one of the main approaches to study structure and properties of CTCs. Computer modeling allows a large number of complexes to be examined in a short period of time to select only a few of the most promising for further experimental research [9].

There have been only a few studies on CTCs with 9,10-phenanthrenequinone nitro derivatives as acceptors [10,11,12]. In [11,12], a series of CTCs based on anthracene, phenanthrene, and 9,10-phenanthrenequinone derivatives was studied. Of all the derivatives considered, 1,3,6-trinitro-9,10-phenanthrenequinone showed the strongest acceptor properties.

**Figure 1:**
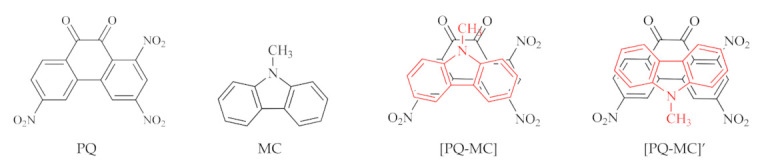
Configurations of acceptor PQ, one of the donors MC, and two possible CTCs [PQ-MC] and [PQ-MC]’.

The purpose of the present work was to study CTCs based on 1,3,6-trinitro-9,10-phenanthrenequinone (PQ) as electron acceptor and different donors. We selected 23 donors with a varying number of π-electrons (from 6 to 26) and a different aromatic system structure; some of them had N and S heteroatoms and substituents. The following donors were used: benzene (BZ), pyridine (PD), N,N,N′,N′-tetramethyl-p-phenylenediamine (TMDA), naphthalene (NA), quinoline (QN), isoquinoline (IQN), acenaphthene (ACN), azulene (AZU), tetrathiafulvalene (TTF), anthracene (AN), phenanthrene (PA), acridine (ACR), 9-methylcarbazole (MC), 5,10-dimethylphenazine (DMPZ), tetracene (TET), tetraphene (TPH), chrysene (CRS), pyrene (PYR), triphenylene (TPL), dibenzotetrathiafulvalene (DBTTF), pentacene (PEN), porphyrin (POR), coronene (COR) (Figure 1 and Supplementary Material Figure S1).

Asymmetrical molecules such as PD, QN, IQN, PA, ACR, CCN, MC, TPH, and AZU produce different stable arrangements of donor and acceptor in CTCs, therefore, the quantum-chemical calculation was performed for 32 possible models (Figure 1 and Figure S2, Table 1).

## 2. Results and Discussion

### 2.1. Theoretical

The formation of charge transfer complexes is controlled by the energy difference (ΔE_MO_) between the LUMO of the isolated acceptor (^A^E_LUMO_) and the HOMO of the isolated donor (^D^E_HOMO_) [3,13]. Since ^A^E_LUMO_ is constant for all the considered CTCs and is equal to −4.57 eV, ΔE_MO_ depends only on ^D^E_HOMO_ which varies from −6.97 to −4.37 eV for selected donors. Based on the ^D^E_HOMO_ values we can expect an increase in donor properties in the following series: PD < BZ < QN < IQN < TPL < NA < PA < ACR < CRS < ACN < COR < PYR < MC < TPH < AN < AZU < POR < TET < DBTTF < PEN < TTF < DMPZ < TMDA (Table 1).

The most important structural features that determine electron donor properties of a molecule are the number and position of the condensed aromatic rings and also the presence of heteroatoms and functional groups. It is evident from Table 1 that donors become stronger as the number of aromatic rings grows. The ΔE_MO_ value decreases in the series of donors with linear arrangement of rings: BZ (2.39 eV), NA (1.47 eV), AN (0.90 eV), TET (0.53 eV), PEN (0.28 eV). The same goes for Δ^CTC^E_MO_: the HOMO–LUMO energy difference in CTCs decreases from 3.10 eV to 1.37 eV for complexes of PQ with benzene and pentacene, respectively.

Δ^CTC^E_MO_ values for anthracene [PQ-AN] (1.83 eV) and phenanthrene [PQ-PA] (2.43 eV)/[PQ-PA]’ (2.57 eV) complexes demonstrate that donors with a linear arrangement of aromatic rings are stronger than those with a non-linear arrangement. Similarly, Δ^CTC^E_MO_ values increase when replacing tetracene [PQ-TET] (1.45 eV) to, tetraphene [PQ-TPH] (1.99 eV), pyrene [PQ-PYR] (2.00 eV), chrysene [PQ-CRS] (2.15 eV), or triphenylene [PQ-TPL] (2.37 eV). Δ^CTC^E_MO_ for the pentacene complex [PQ-PEN] (1.37 eV) is lower than that for coronene [PO-COR] (1.92 eV) (Table 1).

Introduction of a nitrogen heteroatom into donor molecules leads to an increase of Δ^CTC^E_MO_ values for the corresponding complexes, which points to a decrease of donor properties. The same trend for Δ^CTC^E_MO_ holds true when changing BZ for PD, NA for QA/IQA, and AN for ACR (Table 1).

Δ^CTC^E_MO_ values for complexes with acridine (1.88 and 1.72 eV) and azulene (1.57 and 2.10 eV) are found to be less than those of the complex with naphthalene (2.19 eV). Therefore, ACN and AZU are more active donors than NA. It is worth noting that the relative spatial arrangement of AZU and PQ molecules in CTCs (Figure S3) determines not only Δ^CTC^E_MO_ values, but also the formation energies ΔE_ass_ (−63.5 and −73.3 kJ/mol) as well as the mean distance between donor and acceptor planes R (3.16 and 3.11 Å).

Substitution of benzene [PQ-BZ] for tetramethyl-p-phenylenediamine [PQ-TMDA] in the complex changes Δ^CTC^E_MO_ from 3.10 to 1.29 eV. When TTF is replaced with DBTTF Δ^CTC^E_MO_ of the corresponding complexes increases from 1.45 to 1.53 eV. The strongest electron donor in the series considered in this work is DMPZ. Δ^CTC^E_MO_ of [PQ-DMPZ] complex has the lowest value of 1.04 eV.

**Figure 2:**
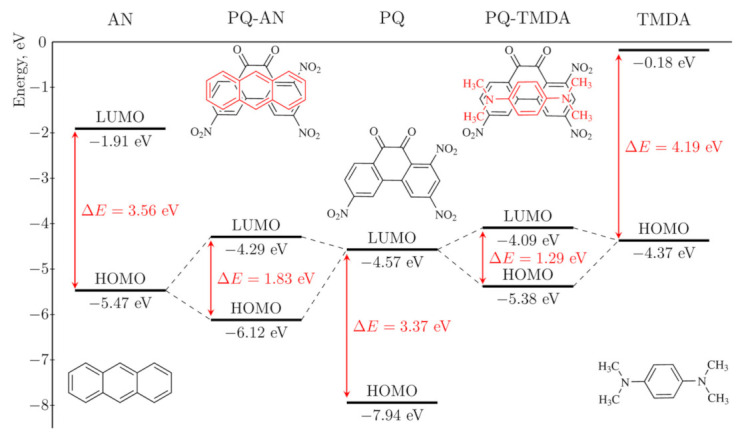
HOMO-LUMO energy levels for complexes of PQ with AN and TMDA according to DFT calculations.

Donor and acceptor orbitals constitute the HOMO and LUMO in complexes (Figure S4). However, upon complexation their energy levels change: ^CTC^E_HOMO_ lies below ^D^E_HOMO_, while ^CTC^E_LUMO_ is higher than ^A^E_LUMO_ (Figure 2). For the CTCs having the highest degree of charge transfer in the series, namely, [PQ-TTF] and [PQ-TMDA], the magnitude of these changes reaches 1.50 eV and 1.49 eV, respectively. As a result, Δ^CTC^E_MO_ values are significantly larger than the corresponding ∆E_MO_ but less than the HOMO-LUMO gaps of isolated PQ or the donors. Figure 2 illustrates this difference for CTCs of PQ with AN and TMDA: Δ^CTC^E_MO_ values (1.83 and 1.29 eV) are smaller than the HOMO-LUMO gaps of PQ (3.37 eV), AN (3.56 eV) and TMDA (4.19 eV). The energy difference values ΔE_MO_ of isolated acceptor and donors in the series decrease from 2.40 to −0.20 eV (Table 1). Notably, ^D^E_HOMO_ of the most pronounced donors, e.g., TTF, TMPZ, and TMDA, lies higher than ^A^E_LUMO_ as graphically exemplified in Figure 2.

The calculated wavelengths of electronic transitions based on the ∆^CTC^E_MO_ values vary from 468 nm for the [PQ-QN]’ complex, to 509 and 481 nm for the [PQ-PA] and [PQ-PA]’ complexes, to 676 nm for the [PQ-AN] complex and further to 959 and 1186 nm for the [PQ-TMDA] and [PQ-DMPZ] complexes, respectively. Two sets of experimentally determined CT absorption bands for complexes [PQ-PA] and [PQ-AN] measured in CH_2_Cl_2_ and toluene are available in the literature. Solutions of complex with phenanthrene absorb at 490 and 495 nm [12], while [PQ-AN] spectra show peaks at 658 and 641 nm for dichloromethane and toluene, respectively [11]. The presence of the charge-transfer bands in the absorption spectra of the complexes is a reliable confirmation of the CTC formation. For a series of CTCs based on the same acceptor, the shift of charge-transfer bands to long wavelengths indicates an increase in donor power. According to this criterion, donor properties increase in the following sequence of molecules: QN, IQN, PA, TPL, NA, ACR, CRS, PYR, TPH, MC, COR, ACN, AZU, AN, POR, DBTTF, TET, TTF, PEN, TMDA, DMPZ.

The amounts of ground state charge transfer in CTCs q_NPA_, calculated as the sum of NPA charges on donor atoms in CTC, is in the range from −0.004 to 0.249 e^−^. Assuming alternating molecular stacking in crystals, these CTCs can be classified as neutral and mixed-valence CT solids [14]. Even a small charge transfer amount of 0.2 is enough for materials to exhibit conducting properties and neutral ionic phase transition in the mixed valent state [14]. For the first excited states the amounts of charge transfer q^*^_NPA_ lie between 0.967 and 1.089 e^−^ (Table 1). This indicates that the electron transitions upon excitation in the CTCs are mainly associated with the transfer of electron density from the donor to the acceptor atoms.

Absolute values of the calculated association energies ∆E_ass_ increase as the π-conjugated system grows. In the series BZ, NA, AN, TET, and PEN ∆E_ass_ values are −47.0, −63.5, −82.3, −106.1, and −118.0 kJ/mol, respectively. The stability of CTCs with TTF, TMDA, DMPZ, POR, and DBTTF is determined not by the size of molecule, but by the presence of heteroatoms (Table 1).

The calculated intermolecular separation distances (R) in CTCs lie in the range from 2.87 to 3.25 Å. The complexes with the lowest distance values exhibit substantial deviation from a planar structure (Figure S5). The calculated R for [PQ-AN] complex (3.24 Å) agrees with the interplanar distance of the X-ray structure (3.49 ± 0.26 Å). The bond lengths of [PQ-AN] predicted by DFT are close to those of both calculated structures of isolated PQ and AN and the X-ray structure (Table 2).

Table S1 demonstrates the NPA charges on atoms of complex [PA-AN] and isolated molecules PQ and AN in both ground and first excited states. In the ground state of [PQ-AN] complex the amount of charge transferred from AN to PQ equals 0.046 e^−^. Interestingly, this value hides the fact that AN carbon atoms gain −0.062 e^−^ while hydrogen atoms lose 0.111 e^−^ when the complex forms. At the same time oxygen and nitrogen atoms are the acceptor centers of PQ.

Upon excitation the charge on the donor increases to 0.990 e^−^ indicating the formation of [D^+^-A^−^] complex. This is accompanied by the electron density transfer from AN carbon atoms to PQ oxygen and nitrogen atoms (0.369 e^−^) and C-atoms (0.624 e^−^).

### 2.2. Experimental

Dark green single prism-shaped crystals of [PQ-AN] complex (I) were grown by slow evaporation from equimolar solution of PQ and AN in CH_2_Cl_2_. The X-ray diffraction study confirmed the 1:1 ratio of PQ and AN in complex I and revealed the monoclinic structure (space group *P*2_1/c_).

The molecular structure of PQ was determined for the first time, although in a complex with AN. It is interesting to discuss and compare the main geometric features of PQ and 2,4,7-trinitro-9,10-phenathrenequinone (TNPQ), especially in complexes with AN (II [11]) and PA (III [12]).

**Figure 3:**
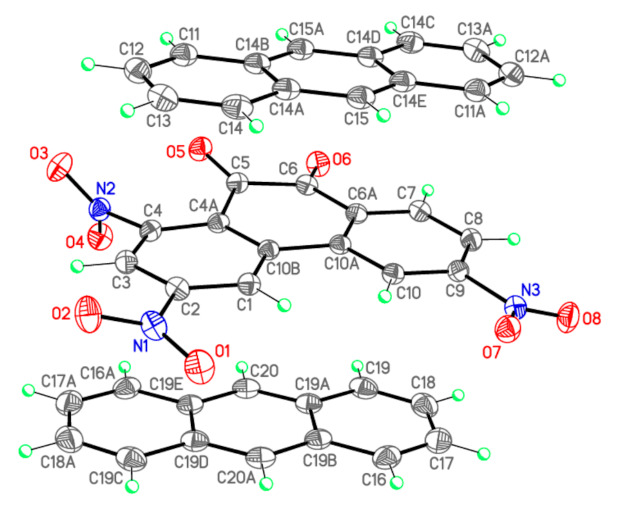
X-ray crystallographic structure of PQ and AN in complex I.

The C=O bond lengths in I (1.2103(13) and 1.2135(13) Å, Table 2, Figure 3) do not differ from those in II (1.211(2) и 1.217(2) Å) [11] and III (1.211(2) и 1.216(2) Å) [12]. The bond C^5^–C^6^ in I (1.5379(15) Å) is significantly longer than the standard single bond of C(*sp*^2^)–C(*sp*^2^) type (1.479 Å) [15]. This deviation is determined by anti-bonding interactions of oxygen atoms in *o*-quinones as was shown earlier in II (1.528(2) Å) [11] and III (1.526(2) Å) [12]. Valence angles of C^5^ and C^6^ in I have values close to those of II and III. In complex I atoms O^5^ и O^6^ are found to deviate notably from the plane of the central ring of PQ (torsion angle O^5^–C^5^–C^6^–O^6^ is 15.54(16)°), which is different from TNPQ where atoms O^5^ and O^6^ lie on the plane of the central ring (0.4(2)° for II [11] and 0.8(2)° for III [12]).

The main structural features of NO_2_ groups in I (Table 2), II [11], and III [12] differ only slightly and are close to the average values [16]: the N–O bond lengths in I are in the range from 1.2198(13) to 1.2289(13) Å and the O–N–O valence angles are from 123.79(10)° to 125.46(10)°. The nitro groups in structure I at atoms C2, C4, and C9 are rotated out of the aromatic plane by 16.84(13), 62.75(13) and 13.56(12)°, respectively, which significantly distinguishes them from similar values at atoms C1, C3, and C8 in II (69.02(19), 0.25(18), and 19.93(18)°) [11] and III (73.8(2), 1.35(15), and 0.95(15)°) [12]. Unlike structures II and III, where the greatest rotation angle was observed in the NO_2_ group at the C^1^ atom, experiencing significant steric repulsion from atoms C^10^ and H^10^, in structure I steric difficulties arise between the nitro group at the atom C^4^ and the carbonyl group O^5^–C^5^, which causes a ~63° rotation of the NO_2_ group and a significant non-planarity of the carbonyl. It should be noted that the C–N bonds near the heavily rotated nitro groups are somewhat elongated relative to other similar bonds: N^2^-C^4^ 1.4791(13) Å in I (Table 2), N^1^-C^1^ 1.480(2) Å in II [11], and N^1^–C^1^ 1.483(2) Å in III [12].

The AN molecule in complex I has C-C bond lengths in the range from 1.359(2) to 1.4400(15) Å and valence angles between 118.32(12)–122.62(11)°, which match with the corresponding values in II (bonds from 1.360(3) to 1.443(2) Å and angles within 118.38(14)−122.50(15)° [11]), in the free AN molecule [15] and in its CTC with 2,3,5,6-tetrachloro-1,4-dicyanobenzene [17].

**Figure 4:**
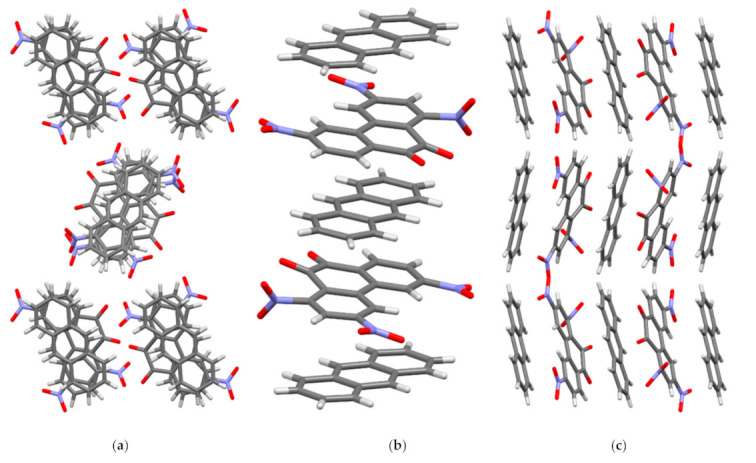
Crystal structure of complex I. (**a**) View along crystallographic *a* axis; (**b**) view along crystallographic *b* axis; (**c**) view along crystallographic *c* axis.

In crystal I, the molecules of the acceptor PQ and the donor AN are arranged parallel to each other and form stacks of mixed type {⋅⋅⋅[A-D]⋅⋅⋅[A-D]’⋅⋅⋅}_∞_ along the crystallographic axis *a* (Figure 4). Every second PQ molecule in the stack is rotated in a plane by 180° relative to the previous one (A and A’), which was observed for TNPQ molecules in II [11]. The AN molecules in I are displaced relative to each other (D and D’) only slightly and their central ring practically overlaps, which distinguishes them significantly from II, where the AN molecules are rotated by 60° relative to each other [11]. As a result of this mutual arrangement of the molecules PQ and AN in I, peculiar triads [D-A]⋅⋅⋅[D-]’ and [D-A]’⋅⋅⋅[D-] are formed, in which the π systems of the donor and the acceptor overlap almost to the same degree (Figure 4).

In I, the PQ and AN molecules form two types of shortened contacts which are less than the sum of the van der Waals radii (Table S2). [A-D] and, [D-A]’ contacts are found in the same stack while A⋅⋅⋅D and A⋅⋅⋅A’ contacts are between the adjacent stacks. In one stack, each acceptor molecule establishes six C⋅⋅⋅C contacts with molecules D and D’ in the range from 3.263(2) to 3.363(2) Å, which may indicate strong π-π interactions between the molecules. The molecules D and D’ form a different number of shortened C⋅⋅⋅C contacts in the stack: each D molecule has four C⋅⋅⋅C contacts with acceptor molecules A and A’, while each D’ has only two such contacts.

The average interplanar distances D⋅⋅⋅A in I are about 3.434(5) Å, which is close to those in CTCs of AN with 2,3,5,6-tetrachloro-1,4-dicyanobenzene (3.427(3) Å) [17] or with 2,3,5,6-tetrafluoro-7,7,8,8-tetracyanoquinodimethane (3.379(2) Å) [18]. The calculated charge transfer values for the CTCs [PQ-AN] (0.046 e^−^) (Table 1) and [TNPQ-AN] (0.091 e^−^) [11] are consistent with the interplanar distances.

Each PQ molecule in I interacts with AN and PQ molecules from adjacent stacks via O⋅⋅⋅H-C shortened contacts in the range of 2.45−2.64 Å. There are also O⋅⋅⋅C contacts between the PQ molecules from adjacent stacks—from 2.921(2) to 3.097(2) Å (Table S2). The presence of such a significant number of various intermolecular interactions in I, the number of which for each PQ molecule reaches twenty-five, and the observed geometric characteristics of PQ can be due to its high acceptor capacity.

## 3. Materials and Methods

### 3.1. Synthesis

1,3,6-trinitro-9,10-phenathrenequinone (PQ, melting point 261−263 °C) was obtained by nitration and subsequent decomposition of 9,10-sulfonyldioxyphenanthrene in concentrated nitric acid (d = 1.51) [19]. Pure-grade anthracene was used without additional purification. The solvent, namely, pure-grade CH_2_Cl_2_ was purified by standard methods. To obtain CTC in the crystalline state, the solutions of acceptor (PQ, 0.2 mmol in 12 mL of CH_2_Cl_2_) and donor (AN, 0.2 mmol in 5 mL of CH_2_Cl_2_) were mixed in equimolar amounts. Single crystals of the [PQ-AN] complex suitable for the X-ray diffraction studies were grown by slow evaporation of the solvent.

### 3.2. X-ray Crystallography and Structure Refinement

The X-ray diffraction study of [PQ-AN] complex was carried out at the “BELOK” beamline of the Kurchatov Institute Synchrotron Radiation Source. The parameters of the unit cell and the reflection intensities were measured using a Rayonix SX165 CCD two coordinate detector (λ = 0.79272 Å, *φ*-scanning in 1.0° steps) (Rayonix LLC, 1880 Oak Ave UNIT 120, Evanston, IL 60201, USA). The structure was solved by direct methods and refined by the full-matrix least squares technique on *F*^2^ with anisotropic displacement parameters for all non-hydrogen atoms using the iMOSFLM (CCP4) [20], SCALA [21], and SHELXL [22] programs. All hydrogen atoms were placed in calculated positions and included in the refinement within the riding model with fixed isotropic displacement parameters *U*_iso_(H) = 1.5*U*_eq_(O), 1.2*U*_eq_(N), and 1.2*U*_eq_(C). The crystallographic data as well as the experimental and refinement parameters are summarized in Table 3. Crystallographic data is available online at the Cambridge Crystallographic Data Centre (CCDC 2099997).

### 3.3. Quantum Chemical Calculations

Quantum chemical simulation of the electronic structure of donor, acceptor, and CTC molecules was performed in the framework of the density functional theory using the B3LYP hybrid functional and the def2-SV(P) basis set. TDDFT methodology was used to explore the low-lying excited states. The Boys–Bernardi method was used for BSSE correction [23]. All D4 dispersion correction was used in all calculations [24]. The amount of charge transfer from a donor to an acceptor was calculated using the natural populations analysis (NPA) [25] as the difference between the sum of charges on the acceptor atoms in free state and in complex for both the ground (Δq_NPA_, e^−^) and first excited (Δq*_NPA_, e^−^) states. The CTC association energies are defined as follows:

ΔE_ass_ = ^CTC^E_tot_ − ^A^E_tot_ − ^D^E_tot_

where ^CTC^E_tot_, ^A^E_tot_ and ^D^E_tot_ are total energies (in kJ/mol) of the CTC, acceptor, and donor, respectively. All the calculations were performed using the Firefly 8.20 software package [26].

## 4. Conclusions

In this study we explored a series of 23 charge transfer complexes based on 1,3,6-trinitro-9,10-phenanthrenequinone and different electron donors by means of density functional theory. Complexes with dibenzotetrathiafulvalene, pentacene, tetrathiafulvalene, 5,10-dimethylphenazine, and tetramethyl-p-phenylenediamine were shown to be in a mixed-valence state with a ground state charge transfer degree of 0.134–0.240 e^−^. A charge transfer complex with anthracene was synthesized, isolated as a single crystal, and the structure determined by X-ray diffraction experiment. Geometric and electronic structure features and their influence on the charge transfer properties of the complexes are discussed.

## Figures and Tables

**Table 1:** The calculated energies (eV) of frontier molecular orbitals of donors and CTCs (^D^E_HOMO_, ^CTC^E_HOMO_, ^CTC^E_LUMO_), energy gaps (eV) of isolated and interacting donor and acceptor (ΔE_MO_, Δ ^CTC^E_MO_), partial NPA charges (e^−^) of complex ground and first excited states (q_NPA_, q*_NPA_), and association energy (ΔE_ass_, kJ/mol).

Molecule	^D^E_HOMO_	ΔE_MO_	^CTC^E_HOMO_	^CTC^E_LUMO_	Δ^CTC^E_MO_	q_NPA_	q*_NPA_	ΔE_ass_
[PQ-PD]	−6.97	2.40	−7.43	−3.92	3.51	0.138	0.080	−59.4
[PQ-Pd]’	−6.97	2.40	−7.61	−4.46	3.14	0.015	0.001	−46.6
[PQ-BZ]	−6.96	2.39	−7.50	−4.40	3.10	0.026	0.008	−47.0
[PQ-QN]	−6.53	1.96	−7.15	−4.35	2.79	0.019	0.071	−67.6
[PQ-Qn]’	−6.53	1.96	−7.06	−4.42	2.64	0.014	0.969	−63.3
[PQ-IQN]	−6.45	1.88	−7.01	−4.45	2.56	−0.003	0.971	−61.4
[PQ-IQn]’	−6.45	1.88	−7.05	−4.45	2.60	0.018	0.967	−64.2
[PQ-TPL]	−6.10	1.53	−6.58	−4.21	2.37	0.037	0.969	−106.5
[PQ-NA]	−6.04	1.47	−6.62	−4.43	2.19	−0.001	0.976	−63.0
[PQ-PA]	−5.98	1.41	−6.65	−4.22	2.43	0.054	0.994	−92.6
[PQ-Pa]	−5.98	1.41	−6.47	−3.89	2.57	0.028	0.992	−89.9
[PQ-ACR]	−5.92	1.35	−6.48	−4.30	2.18	0.014	0.982	−79.2
[PQ-ACr]’	−5.92	1.35	−6.50	−4.38	2.11	0.023	0.981	−77.1
[PQ-CRS]	−5.76	1.19	−6.36	−4.21	2.15	0.060	1.007	−111.3
[PQ-ACN]	−5.71	1.14	−6.31	−4.43	1.88	0.005	0.983	−74.7
[PQ-aCn]’	−5.71	1.14	−6.25	−4.53	1.72	0.047	0.985	−75.8
[PQ-COR]	−5.70	1.13	−6.13	−4.21	1.92	0.031	1.010	−115.3
[PQ-PYR]	−5.58	1.01	−6.20	−4.20	2.00	0.068	0.992	−97.5
[PQ-MC]	−5.57	1.00	−6.27	−4.31	1.96	0.070	1.012	−94.0
[PQ-Mc]’	−5.57	1.00	−6.16	−4.23	1.92	0.071	1.008	−87.0
[PQ-TPH]	−5.57	1.00	−6.17	−4.18	1.99	0.066	1.006	−104.8
[PQ-TPh]’	−5.57	1.00	−6.15	−4.21	1.94	0.065	1.005	−107.0
[PQ-AN]	−5.47	0.90	−6.12	−4.29	1.83	0.046	0.990	−82.3
[PQ-AZU]	−5.44	0.87	−6.02	−4.45	1.57	−0.004	0.969	−63.5
[PQ-AZu]’	−5.44	0.87	−6.27	−4.18	2.10	0.114	0.971	−73.3
[PQ-POR]	−5.39	0.82	−5.88	−4.07	1.82	0.077	1.033	−119.2
[PQ-TET]	−5.10	0.53	−5.70	−4.24	1.45	0.066	1.018	−106.1
[PQ-DBTTF]	−4.89	0.32	−5.53	−4.00	1.53	0.216	1.081	−124.0
[PQ-PEN]	−4.85	0.28	−5.45	−4.08	1.37	0.134	1.042	−118.0
[PQ-TTF]	−4.52	−0.05	−5.48	−4.03	1.45	0.240	1.089	−102.3
[PQ-DMPZ]	−4.39	−0.18	−5.26	−4.22	1.04	0.135	1.058	−107.5
[PQ-TMDA]	−4.37	−0.20	−5.38	−4.09	1.29	0.224	1.075	−104.3

**Table 2:** Bond lengths *d* (Å) and valence angles ω (deg.) of complex I (X-ray diffraction data), complex [PQ-AN] and isolated PQ and AN molecules (DFT calculations). Atom numbering scheme is given in Figure 3.

Bond	*d*			Angle	ω		
	I	[PQ-AN]	PQ, AN		I	[PQ-AN]	PQ, AN
O^5^–C^5^	1.2103(13)	1.208	1.207	O^1^–N^1^–O^2^	124.64(10)	125.5	125.9
O^6^–C^6^	1.2135(13)	1.211	1.209	O^2^–N^1^–C^2^	117.20(9)	117.1	117.0
O^1^–N^1^	1.2258(13)	1.219	1.217	O^1^–N^1^–C^2^	118.16(9)	117.4	117.1
O^2^–N^1^	1.2257(13)	1.218	1.217	O^3^–N^2^–O^4^	125.46(10)	126.7	127.2
O^3^–N^2^	1.2198(13)	1.214	1.215	O^4^–N^2^–C^4^	117.13(9)	116.4	116.4
O^4^–N^2^	1.2211(13)	1.217	1.214	O^3^–N^2^–C^4^	117.32(9)	116.7	116.3
O^7^–N^3^	1.2289(13)	1.220	1.218	O^7^–N^3^–O^8^	123.79(10)	125.2	125.6
O^8^–N^3^	1.2264(13)	1.219	1.217	O^7^–N^3^–C^9^	118.31(9)	117.4	117.3
N^1^–C^2^	1.4712(14)	1.479	1.485	O^8^–N^3^–C^9^	117.89(9)	117.4	117.1
N^2^–C^4^	1.4791(13)	1.480	1.482	O^5^–C^5^–C^4A^	122.52(10)	123.0	122.8
N^3^–C^9^	1.4717(13)	1.477	1.484	O^5^–C^5^–C^6^	119.50(10)	120.0	119.8
C^5^–C^6^	1.5379(15)	1.537	1.540	C^4A^–C^5^–C^6^	117.91(9)	117.0	117.3
C^10A^–C^10B^	1.4819(15)	1.480	1.484	O^6^–C^6^–C^6A^	123.53(10)	123.3	123.2
C^11^–C^12^	1.3606(18)	1.373	1.373	O^6^–C^6^–C^5^	118.92(10)	119.6	119.5
C^12^–C^13^	1.4213(17)	1.427	1.428	C^6A^–C^6^–C^5^	117.54(9)	117.1	117.2
C^11^–C^14B^	1.4303(17)	1.431	1.431	C^3^–C^4^–N^2^	115.79(9)	115.7	115.9
C^14A^–C^15^	1.3968(17)	1.403	1.402	C^4A^–C^4^–N^2^	120.83(9)	121.4	121.4
C^14A^–C^14B^	1.4400(15)	1.446	1.446	C^12^–C^11^–C^14B^	120.73(11)	120.8	121.0

**Table 3:** Summary of crystallographic experiment data and structure refinement parameters for I.

Compound	I
CCDC	2099997
Formula	C_14_H_5_N_3_O_8_·C_14_H_10_
Crystal system	Monoclinic
Space group	*P*2_1/c_
Z	4
*a*,*b*,*c*, Å	14.2721(14), 19.479(2), 8.1900(9)
α, β, γ, deg	90, 99.041(8), 90
*V*, Å^3^	2248.6(4)
*D_x_*, g/cm^3^	1.540
Radiation, λ, Å	Synchrotron, 0.79272
*μ*, mm^−1^	0.147
*T*, K	100(2)
Specimen size, mm	0.18 × 0.15 × 0.03
Absorption correction	Semi-empirical
*T*_min_/*T*_max_	0.966/0.987
*θ*_max_, deg	30.95
Limits of *h*,*k*,*l*	−15<=h<=18; −25<=k<=18; −10<=l<=10;
Number of reflections: measured/independent (*N*_1_);	13943/5033
observed with *I*>2σ(*I*) (*N*_2_)	4596
*R*_*int*_	0.0276
Number of parameters	353
Extinction coefficient	0.029(3)
*R*_1_/*wR*_2_ by *N*_1_	0.0404/0.0975
*R*_1_/*wR*_2_ by *N*_2_	0.0373/0.0950
*S*	1.023
Δ*p*_min_/Δ*p*_max_, eÅ^−3^	−0.220/0.261

## Data Availability

Not applicable.

## Supplementary Materials

The following are available online. Figure S1: Chemical structure depiction of PQ and 23 donors used in this work, Figure S2: Configurations of charge transfer complexes considered in this work, Figure S3: DFT optimized molecular structure of complexes: (a) [PQ-AZU] and (b) [PQ-AZu]’, Figure S4: Electron density corresponding to (a) HOMO and (b) LUMO of [PQ-AN] complex, Figure S5: DFT optimized molecular structure of complexes: (a) [PQ-DBTTF] (R = 2.87 Å) and (b) [PQ-DMPZ] (R = 3.02 Å), Table S1: Calculated NPA partial charges on atoms of complex [PQ-AN] in ground and first ex-cited ([PQ-AN]*) states and of isolated PQ and AN molecules, Table S2: Selected shortened contacts d (Å) between PQ and AN in complex I.

## Sample Availability

Samples of the compounds are not available from the authors.

## Abbreviations

ΔE_MO_	the energy difference between ^A^E_LUMO_ and ^D^E_HOMO_
^D^E_HOMO_	energy level of the HOMO of isolated donor molecule
^A^E_LUMO_	energy level of the LUMO of isolated acceptor molecule
Δ^CTC^E_MO_	the energy difference between ^CTC^E_LUMO_ and ^CTC^E_HOMO_
^CTC^E_HOMO_	energy level of the HOMO of complex
^CTC^E_LUMO_	energy level of the LUMO of complex
q_NPA_	partial NPA charges in the ground state
q*_NPA_	partial NPA charges in the first excited state
ΔE_ass_	energy of association of donor and acceptor
A	acceptor
ACN	acenaphthene
ACR	acridine
AN	anthracene
AZU	azulene
BZ	benzene
COR	coronene
CRS	chrysene
CTCs	charge transfer complexes
D	donor
DBTTF	dibenzotetrathiafulvalene
DFT	density functional theory
DMPZ	5,10-dimethylphenazine
HOMO	highest occupied molecular orbital
IQN	isoquinoline
LUMO	lowest unoccupied molecular orbital
MC	9-methylcarbazole
NA	naphthalene
PA	phenanthrene
PD	pyridine
PEN	pentacene
POR	porphyrin
PQ	1,3,6-trinitro-9,10-phenanthrenequinone
PYR	pyrene
QN	quinoline
TET	tetracene
TMDA	N,N,N’,N’-tetramethyl-p-phenylenediamine
TPH	tetraphene
TPL	triphenylene
TTF	tetrathiafulvalene
