# Supplementary material for: Charge Transfer Complexes of 1,3,6-Trinitro-9,10-phenanthrenequinone with Polycyclic Aromatic Compounds
Source: Molecules. 2021 Oct 22;26(21):6391. doi: 10.3390/molecules26216391 (PMC8588456; doi:10.3390/molecules26216391)

checkCIF/PLATON report

Structure factors have been supplied for datablock(s) I

THIS REPORT IS FOR GUIDANCE ONLY. IF USED AS PART OF A REVIEW PROCEDURE FOR PUBLICATION, IT SHOULD NOT REPLACE THE EXPERTISE OF AN EXPERIENCED CRYSTALLOGRAPHIC REFEREE.

No syntax errors found. CIF dictionary Interpreting this report

Datablock: I

```
Bond precision:  C-C = 0.0016 A          Wavelength=0.79272

Cell:            a=14.2721(14)     b=19.479(2)        c=8.1900(9)
                 alpha=90          beta=99.041(8)     gamma=90
Temperature:     100 K

                Calculated                 Reported
Volume          2248.6(4)                  2248.6(4)
Space group     P 21/c                     P 21/c
Hall group      -P 2ybc                    -P 2ybc
Moiety formula  C14 H5 N3 O8, C14 H10      C14 H5 N3 O8, C14 H10
Sum formula     C28 H15 N3 O8              C28 H15 N3 O8
Mr              521.43                     521.43
Dx,g cm-3       1.540                      1.540
Z               4                          4
Mu (mm-1)       0.147                      0.147
F000            1072.0                     1072.0
F000'           1072.84
h,k,lmax        18,25,10                   18,25,10
Nref            5152                       5033
Tmin,Tmax       0.974,0.996                0.966,0.987
Tmin'           0.974

Correction method= # Reported T Limits: Tmin=0.966 Tmax=0.987
AbsCorr = MULTI-SCAN

Data completeness= 0.977          Theta(max)= 30.950

R(reflections)= 0.0373( 4596)     wR2(reflections)= 0.0975( 5033)

S = 1.023                  Npar= 353
```

The following ALERTS were generated. Each ALERT has the format
test-name_ALERT_alert-type_alert-level.
Click on the hyperlinks for more details of the test.

Alert level C

```
PLAT029_ALERT_3_C _diffrn_measured_fraction_theta_full value Low .      0.977 Why?
PLAT369_ALERT_2_C Long   C(sp2)-C(sp2) Bond  C5       - C6       .       1.54 Ang.
PLAT410_ALERT_2_C Short Intra H...H Contact  H1       ..H10      .       1.98 Ang.
                                                       x,y,z  =      1_555 Check
PLAT910_ALERT_3_C Missing # of FCF Reflection(s) Below Theta(Min).          6 Note
PLAT911_ALERT_3_C Missing FCF Refl Between Thmin & STh/L=    0.600         89 Report
PLAT913_ALERT_3_C Missing # of Very Strong Reflections in FCF ....         18 Note
```

Alert level G

```
ABSMU01_ALERT_1_G  Calculation of _exptl_absorpt_correction_mu
                not performed for this radiation type.
PLAT092_ALERT_4_G Check: Wavelength Given is not Cu,Ga,Mo,Ag,In Ka    0.79272 Ang.
PLAT432_ALERT_2_G Short Inter X...Y Contact  O7       ..C5               2.92 Ang.
                                                     x,y,1+z  =      1_556 Check
PLAT912_ALERT_4_G Missing # of FCF Reflections Above STh/L=  0.600         24 Note
PLAT933_ALERT_2_G Number of OMIT Records in Embedded .res File ...         10 Note
PLAT941_ALERT_3_G Average HKL Measurement Multiplicity ...........        2.8 Low
PLAT978_ALERT_2_G Number C-C Bonds with Positive Residual Density.         19 Info
```

0 **ALERT level A** = Most likely a serious problem - resolve or explain
0 **ALERT level B** = A potentially serious problem, consider carefully
6 **ALERT level C** = Check. Ensure it is not caused by an omission or oversight
7 **ALERT level G** = General information/check it is not something unexpected

1 ALERT type 1 CIF construction/syntax error, inconsistent or missing data
5 ALERT type 2 Indicator that the structure model may be wrong or deficient
5 ALERT type 3 Indicator that the structure quality may be low
2 ALERT type 4 Improvement, methodology, query or suggestion
0 ALERT type 5 Informative message, check

It is advisable to attempt to resolve as many as possible of the alerts in all categories. Often the minor alerts point to easily fixed oversights, errors and omissions in your CIF or refinement strategy, so attention to these fine details can be worthwhile. In order to resolve some of the more serious problems it may be necessary to carry out additional measurements or structure refinements. However, the purpose of your study may justify the reported deviations and the more serious of these should normally be commented upon in the discussion or experimental section of a paper or in the "special_details" fields of the CIF. checkCIF was carefully designed to identify outliers and unusual parameters, but every test has its limitations and alerts that are not important in a particular case may appear. Conversely, the absence of alerts does not guarantee there are no aspects of the results needing attention. It is up to the individual to critically assess their own results and, if necessary, seek expert advice.

Publication of your CIF in IUCr journals

A basic structural check has been run on your CIF. These basic checks will be run on all CIFs submitted for publication in IUCr journals (*Acta Crystallographica*, *Journal of Applied Crystallography*, *Journal of Synchrotron Radiation*); however, if you intend to submit to *Acta Crystallographica Section C* or *E* or *IUCrData*, you should make sure that full publication checks are run on the final version of your CIF prior to submission.

Publication of your CIF in other journals

Please refer to the *Notes for Authors* of the relevant journal for any special instructions relating to CIF submission.

PLATON version of 13/07/2021; check.def file version of 13/07/2021

Datablock I - ellipsoid plot

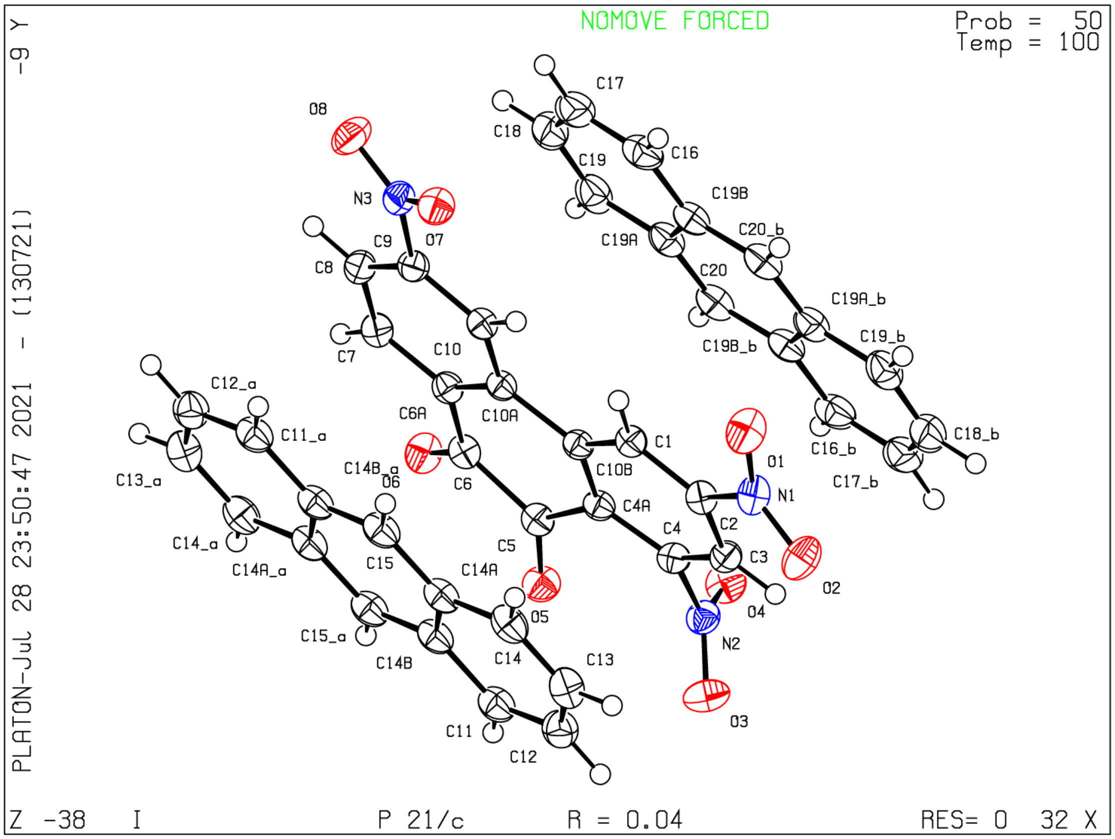

Supplement: Supplementary file 1 [file molecules-26-06391-s001.zip › I_checkcif.pdf]
