# Supplementary material for: Charge Transfer Complexes of 1,3,6-Trinitro-9,10-phenanthrenequinone with Polycyclic Aromatic Compounds
Source: Molecules. 2021 Oct 22;26(21):6391. doi: 10.3390/molecules26216391 (PMC8588456; doi:10.3390/molecules26216391)
Supplement: Supplementary file 1 [file molecules-26-06391-s001.zip › Molecules_ESI_Linko.pdf]

molecules

MDPI

Electronic Supplementary Information

Charge Transfer Complexes of 1,3,6-trinitro-9,10-phenanthrenequinone with Polycyclic Aromatic Compounds

Roman Linko, Michael Ryabov, Pavel Strashnov, Pavel Dorovatovskii, Victor Khrustalev and Victor Davydov

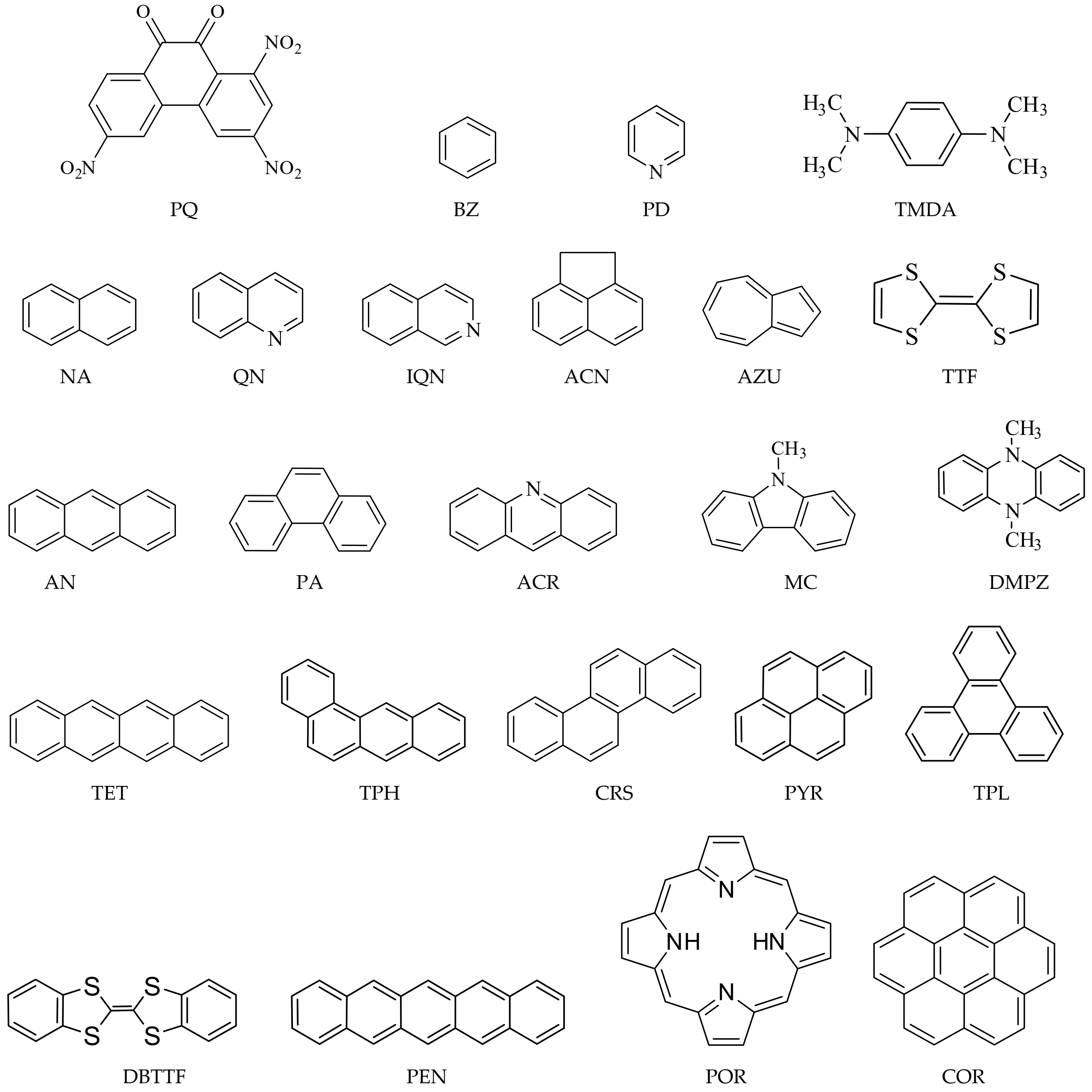

Figure S1. Chemical structure depiction of PQ and 23 donors used in this work.

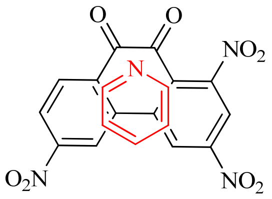
[PQ-PD]

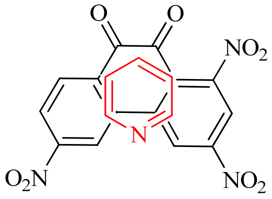
[PQ-PD]′

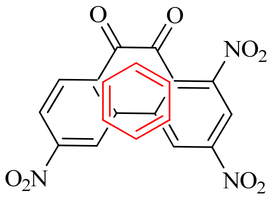
[PQ-BZ]

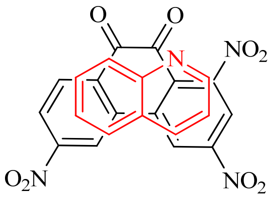
[PQ-QN]

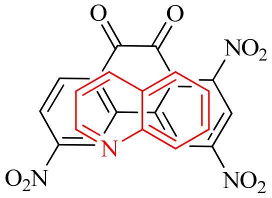
[PQ-QN]′

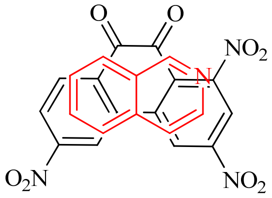
[PQ-IQN]

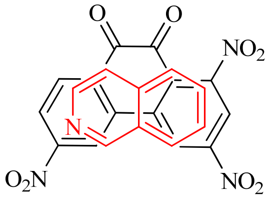
[PQ-IQN]′

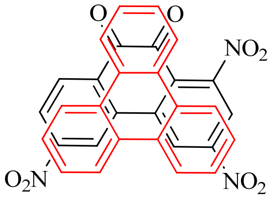
[PQ-TPL]

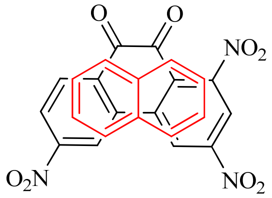
[PQ-NA]

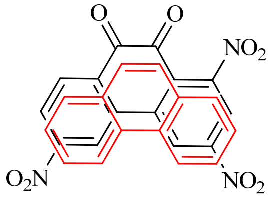
[PQ-PA]

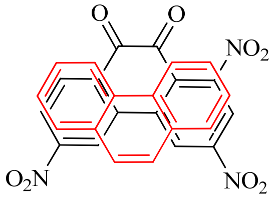
[PQ-PA]′

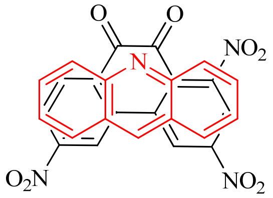
[PQ-ACR]

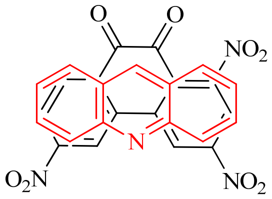
[PQ-ACR]′

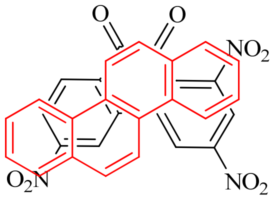
[PQ-CRS]

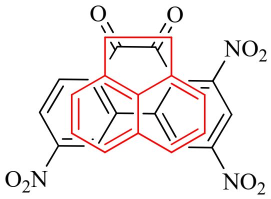
[PQ-ACN]

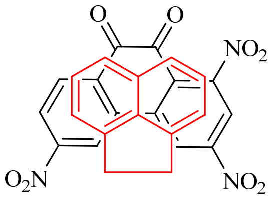
[PQ-ACN]′

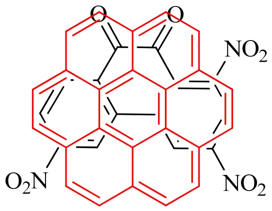
[PQ-COR]

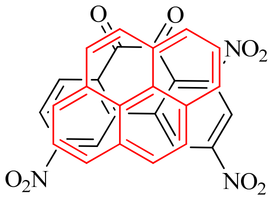
[PQ-PYR]

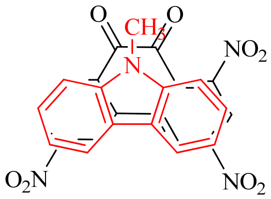
[PQ-MC]

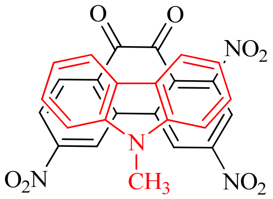
[PQ-MC]′

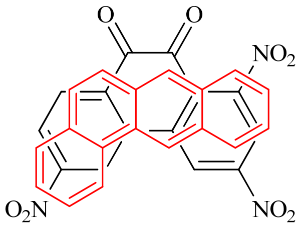
[PQ-TPH]

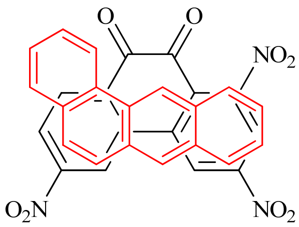
[PQ-TPH]′

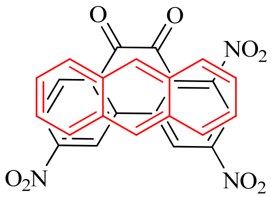
[PQ-AN]

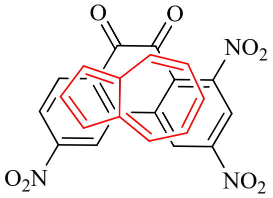
[PQ-AZU]

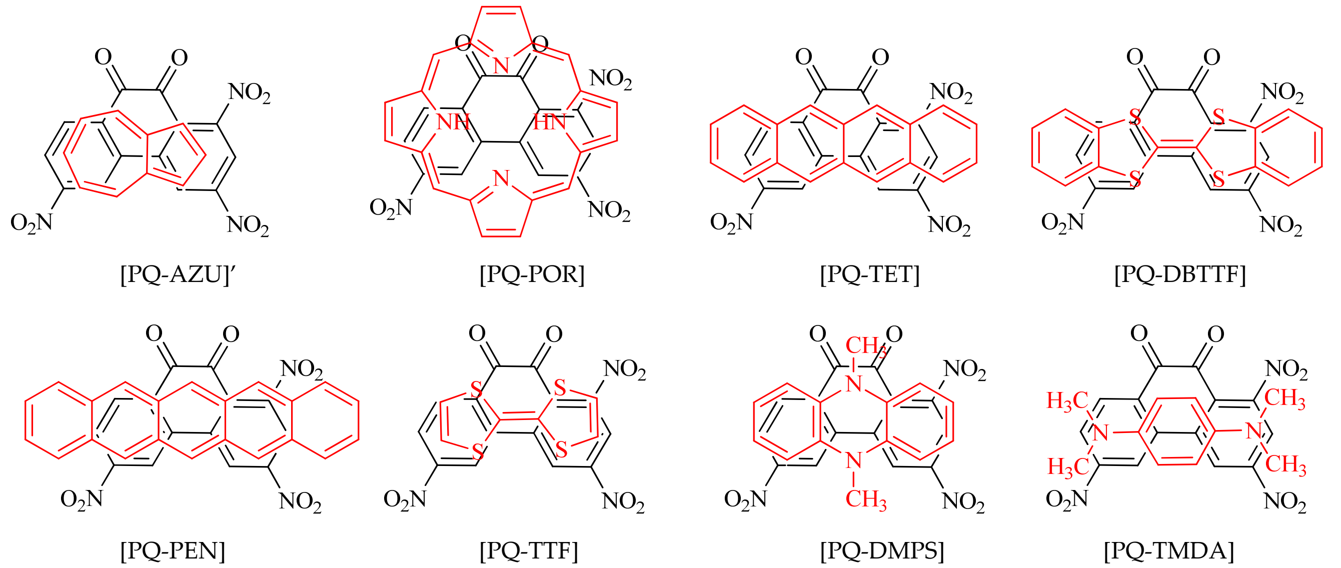

Figure S2. Configurations of charge transfer complexes considered in this work.

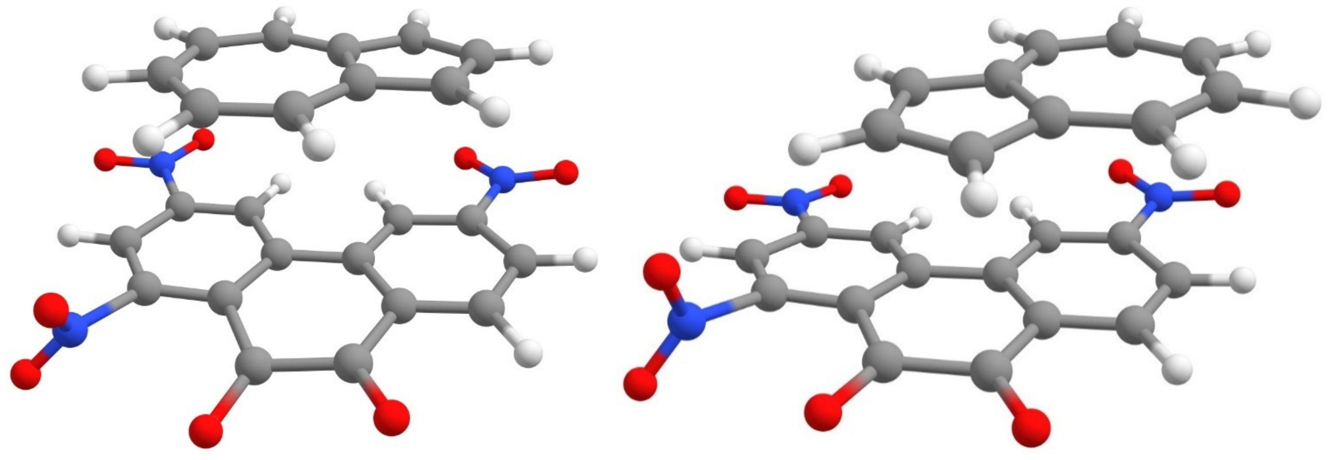

(**a**) (**b**)

Figure S3. DFT optimized molecular structure of complexes: (a) [PQ-AZU] and (b) [PQ-AZU]′.

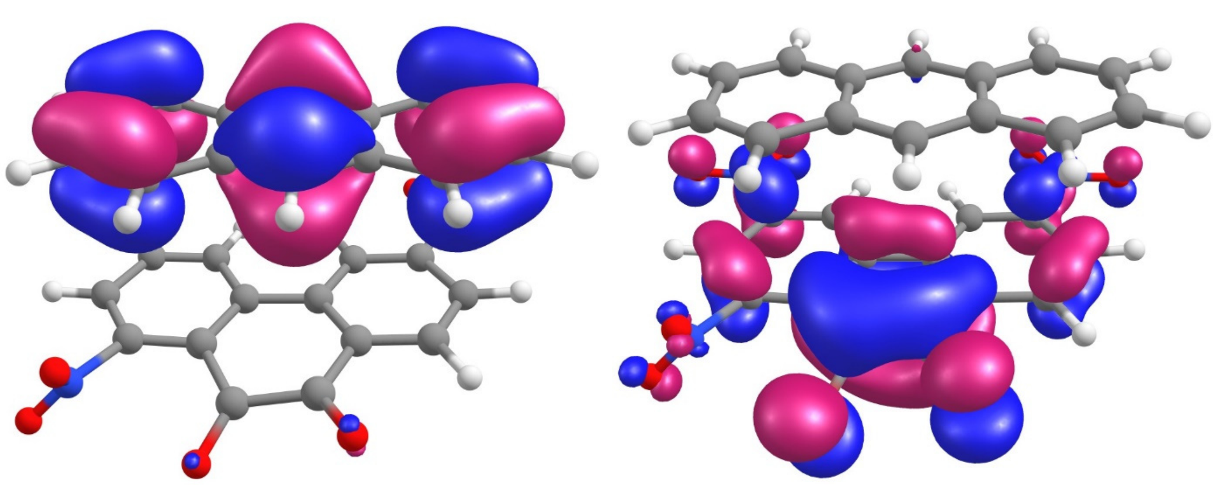

(a) **(b)**

Figure S4. Electron density corresponding to **(a)** HOMO and **(b)** LUMO of [PQ-AN] complex.

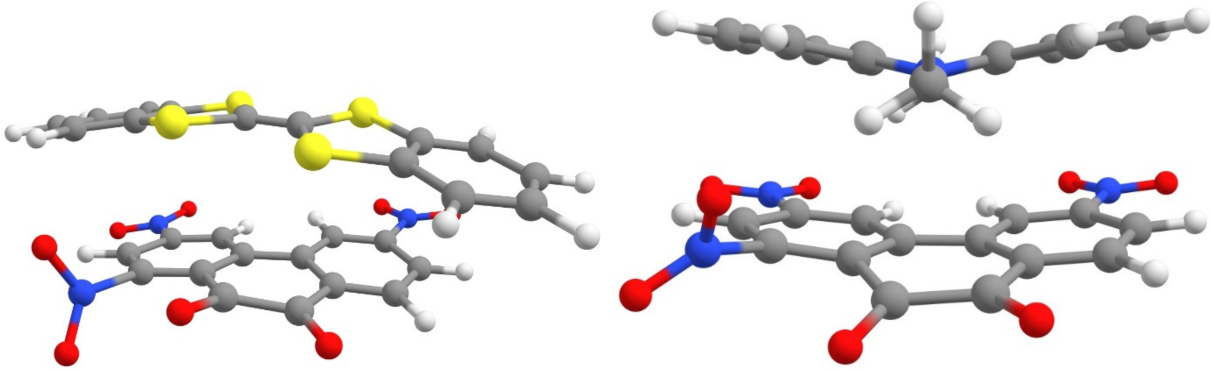

(a) **(b)**

Figure S5. DFT optimized molecular structure of complexes: **(a)** [PQ-DBTTF] (*R* = 2,87 Å) and **(b)** [PQ-DMPZ] (*R* = 3,02 Å).

Table S1. Calculated NPA partial charges on atoms of complex [PQ-AN] in ground and first excited ([PQ-AN]*) states and of isolated PQ and AN molecules (numerating scheme is given in fig. 3)

Atom	[PQ-AN]	[PQ-AN]*	PQ	Atom	[PQ-AN]	[PQ-AN]*	AN
O^{1}	-0.372	-0.406	-0.365	C^{11}	-0.194	-0.077	-0.185
O^{2}	-0.369	-0.401	-0.359	C^{12}	-0.216	-0.146	-0.214
O^{3}	-0.343	-0.350	-0.346	C^{13}	-0.212	-0.176	-0.214
O^{4}	-0.357	-0.361	-0.340	C^{14}	-0.185	-0.079	-0.185
O^{5}	-0.470	-0.597	-0.459	C^{14A}	-0.065	-0.084	-0.062
O^{6}	-0.487	-0.592	-0.474	C^{14B}	-0.069	-0.078	-0.062
O^{7}	-0.383	-0.384	-0.371	C^{15}	-0.167	+0.038	-0.158
O^{8}	-0.373	-0.397	-0.364	C^{15A}	-0.154	+0.043	-0.158
N^{1}	+0.519	+0.495	+0.514	C^{14D}	-0.070	-0.077	-0.062

N^{2}	+0.518	+0.515	+0.515	C^{14E}	-0.068	-0.074	-0.062
N^{3}	+0.517	+0.509	+0.513	C^{14C}	-0.194	-0.090	-0.185
C^{1}	-0.162	-0.161	-0.166	C^{13A}	-0.187	-0.166	-0.214
C^{2}	+0.109	+0.044	+0.104	C^{12A}	-0.222	-0.164	-0.214
C^{3}	-0.185	-0.212	-0.186	C^{11A}	-0.219	-0.103	-0.185
C^{4}	+0.148	+0.116	+0.146	$H(C^{11})$	+0.233	+0.228	+0.215
C^{4A}	-0.115	-0.188	-0.113	$H(C^{12})$	+0.226	+0.223	+0.217
C^{5}	+0.500	+0.394	+0.500	$H(C^{13})$	+0.230	+0.227	+0.217
C^{6}	+0.500	+0.415	+0.501	$H(C^{14})$	+0.227	+0.222	+0.215
C^{6A}	-0.117	-0.153	-0.118	$H(C^{15})$	+0.224	+0.216	+0.214
C^{7}	-0.145	-0.168	-0.141	$H(C^{15A})$	+0.223	+0.215	+0.214
C^{8}	-0.175	-0.206	-0.183	$H(C^{14C})$	+0.224	+0.220	+0.215
C^{9}	+0.096	+0.066	+0.097	$H(C^{13A})$	+0.223	+0.221	+0.217
$C^{1}0$	-0.168	-0.163	-0.171	$H(C^{12A})$	+0.229	+0.227	+0.217
$C^{10}A$	-0.028	-0.056	-0.031	$H(C^{11A})$	+0.228	+0.225	+0.215
C^{10B}	-0.003	-0.049	-0.011				
$H(C^{1})$	+0.259	+0.259	+0.258				
$H(C^{3})$	+0.277	+0.277	+0.280				
$H(C^{7})$	+0.249	+0.249	+0.252				
$H(C^{8})$	+0.260	+0.261	+0.262				
$H(C^{10})$	+0.254	+0.254	+0.254				

Table S2. Selected shortened contacts *d* (Å) between **PQ** and **AN** in complex **I** (X-ray data, numerating scheme is given in fig. 3)

Contacts in one stack	*d*	Contacts between stacks	*d*
[A—D]		A···D	
$C^{5}\cdots C^{15}$	3.263(2)	$O^{3}\cdots H^{13}—C^{13}$	2.61
$C^{5}\cdots C^{14B}$	3.363(2)	$O^{3}\cdots H^{14}—C^{14}$	2.64
$C^{6}\cdots C^{15}$	3.284(2)	$O^{5}\cdots H^{11}—C^{11}$	2.53
$C^{6A}\cdots C^{14A}$	3.316(2)	$O^{5}\cdots H^{15}—C^{15}$	2.45
		$O^{7}\cdots H^{20}—C^{20}$	2.48
[A—D]′			
$C^{2}\cdots C^{19A}$	3.306(2)		
$C^{9}\cdots C^{18}$	3.342(2)		
		A···A	
$C^{3}—H^{3}\cdots O^{2}$	2.45	$O^{2}\cdots H^{3}—C^{3}$	2.45
$C^{1}—H^{1}\cdots O^{4}$	2.51	$O^{4}\cdots H^{1}—C^{1}$	2.51
$C^{10}—H^{10}\cdots O^{4}$	2.53	$O^{4}\cdots H^{10}—C^{10}$	2.53
$C^{5}\cdots O^{7}$	2.921(2)	$O^{7}\cdots C^{5}$	2.921
$C^{6}\cdots O^{7}$	3.081(2)	$O^{7}\cdots C^{6}$	3.081(2)
$C^{7}\cdots O^{8}$	3.067(2)	$O^{8}\cdots C^{7}$	3.067(2)
$C^{8}\cdots O^{8}$	3.097(2)	$O^{8}\cdots C^{8}$	3.097(2)
